# Comparison of dental implant placement accuracy between robotic and static or dynamic computer-assisted surgeries: A systematic review and meta-analysis

**DOI:** 10.4317/medoral.27132

**Published:** 2025-10-17

**Authors:** Jiawen Wang, Mengqi Gao, Yaoyu Zhao, Bin Shi, Xinyu Wu, Yufeng Zhang, Qi Yan

**Affiliations:** 1 School of Medicine, JingChu University of Technology; 2 State Key Laboratory of Oral & Maxillofacial Reconstruction and Regeneration, Key Laboratory of Oral Biomedicine Ministry of Education, Hubei Key Laboratory of Stomatology, School & Hospital of Stomatology, Wuhan University; 3 Department of Oral Implantology, School & Hospital of Stomatology, Wuhan University, Wuhan, China; 4 Department of Oral and Maxillofacial Implantology, Shanghai PerioImplant Innovation Center, Shanghai Ninth People's Hospital, Shanghai Jiao Tong University School of Medicine, College of Stomatology, Shanghai Jiao Tong University, National Center for Stomatology, National Clinical Research Center for Oral Diseases, Shanghai Key Laboratory of Stomatology, Shanghai Research Institute of Stomatology; 5 Medical Research Institute, School of Medicine, Wuhan University

## Abstract

**Background:**

Robotic computer-assisted implant surgery (rCAIS) has been developed to enhance implant placement accuracy compared to static (sCAIS) and dynamic (dCAIS) computer-assisted implant surgeries. The aim of this systematic review and meta-analysis aimed to evaluate and compare the accuracy of rCAIS to other CAIS approaches.

**Material and Methods:**

Electronic searches were conducted in PubMed, Embase, Cochrane Library and CNKI up to September 2024. Additionally, a manual search of relevant journals and reference lists was performed. Clinical and preclinical studies comparing rCAIS with sCAIS or dCAIS were included. Primary outcomes were global platform deviation, global apex deviation, and angular deviation between planned and actual implant positions. Two independent reviewers extracted data and assessed risk of bias using RoB 2 for randomized trials, the ROBINS-I tool for non-randomized studies, the SYRCLE tool for animal studies, and the QUIN tool for in vitro studies.

**Results:**

Eleven studies met the inclusion criteria. In clinical studies, rCAIS (157 implants) demonstrated significantly lower deviations compared to sCAIS (166 implants): Global platform MD=-0.73mm (95% CI: -1.00 to -0.45; p&lt;0.00001), apex deviation MD=-0.84mm (95% CI: -1.12 to -0.56; p&lt;0.00001), and angular deviation MD=-1.51° (95% CI: -2.71 to -0.32; p=0.01). In preclinical studies, rCAIS also outperformed dCAIS (both 270 implants): Platform deviation MD=-0.15mm (95% CI: -0.24 to -0.07; p=0.0002), apex deviation MD=-0.19mm (95% CI: -0.27 to -0.10; p&lt;0.0001), and angular deviation MD=-1.03° (95% CI: -1.70 to -0.37; p=0.002).

**Conclusions:**

rCAIS demonstrates superior accuracy compared to sCAIS or dCAIS. However, the magnitude of observed differences is small, and thus the improvements may not be clinically relevant despite the statistical significance. Further well-designed and large-scale studies are warranted to explore the influencing factors and optimize the clinical application of rCAIS.

## Introduction

The accurate three-dimensional position of dental implants is essential for achieving both aesthetic and functional restoration, ensuring the long-term health of the implant (1). In traditional freehand implant surgeries, implant placement accuracy depends on the surgeon's expertise, which introduces variability (2). Computer-assisted implant surgeries (CAIS), including static CAIS (sCAIS) and dynamic CAIS (dCAIS), have been integrated into routine clinical practice to reduce the risk of surgical complications.

sCAIS could lead to implant positioning errors of about one millimeter and angulation errors up to 3° (3), while dCAIS also provides clinically acceptable accuracy (4). However, both methods have limitations. sCAIS faces challenges such as restricted mouth opening (5), limited cooling, and a fixed treatment plan without real-time adjustment (6). dCAIS depends heavily on the surgeon's skill, with the lack of haptic feedback requiring constant focus on the screen, creating a steep learning curve (6).

With advances in technology, robotic CAIS (rCAIS) has emerged to overcome the limitations of traditional CAIS methods. The first FDA-approved robot for edentulous arches exhibited high precision in implant placement. A recent case series by Yang et al. (7) report promising accuracy and safety levels. While numerous studies have assessed rCAIS accuracy, few have directly compared it to sCAIS or dCAIS. Takács et al. reviewed single-arm studies (8), Khaohoen et al. focused on clinical trials (9) and Jain et al. examined only in vitro studies (10).

This study aims to compare the accuracy of rCAIS with both sCAIS and dCAIS in clinical and preclinical settings, using available double-arm studies. The null hypothesis is that there is no statistically significant difference in implant placement accuracy between rCAIS and sCAIS or between rCAIS and dCAIS.

## Material and Methods

This study followed the PRISMA 2020 statements (11) for systematic reviews and meta-analyses. Ethics approval was not required as no human or animal studies were included. The study protocol was prospectively registered with PROSPERO (No. CRD42023469332).

Eligibility criteria

The PICOS question was defined as follows. Population (P): Partially or completely edentulous participants. This includes clinical human subjects as well as preclinical models, such as in vitro studies and animal experiments. Intervention (I): Robot-assisted computer-assisted implant surgery (rCAIS). Comparison (C): Static computer-assisted implant surgery (sCAIS) or dynamic computer-assisted implant surgery (dCAIS). Outcome (O): Accuracy of dental implant placement, assessed using parameters such as angular deviation, global coronal deviation, and global apical deviation. Study Design (S): Prospective and retrospective studies, including randomized controlled trials (RCTs), cohort studies, case-control studies and preclinical studies that compare rCAIS with either sCAIS or dCAIS.

Moreover, studies were included and excluded based on the following criteria.

Inclusion criteria: Studies involving implants placed in edentulous or partially edentulous participants as well as preclinical models (in vitro studies, animal studies and cadaveric studies). Studies with more than ten implants placed using rCAIS (including pilot studies). Studies directly comparing the accuracy of implants placed with rCAIS to sCAIS or dCAIS. Studies reporting global platform deviation, global apex deviation, and angular deviation on CBCT.

Exclusion criteria: Studies without original data (e.g., reviews, case reports, editorials). Studies involving zygomatic implants. Studies published in languages other than English or Chinese.

Information sources and search strategy

The electronic databases searched included PubMed, Embase, Cochrane Library, and CNKI, with the search conducted on September 19, 2024. The search strategy used keywords such as "implants", "robot", "static", "dynamic", "assist", "accuracy", and their synonyms. A detailed search strategy is provided in Table 1. Additionally, a manual search of the reference lists from related systematic reviews and studies was conducted.

Table 1

Study selection and data collection process

Two authors (J. W. and M. G.) independently screened titles and abstracts using Zotero reference manager (version 7.0.0). Inter-examiner reliability was calculated with the Cohen kappa statistic (). Full texts of relevant articles were obtained, and studies not meeting inclusion criteria were documented with reasons for exclusion. Discrepancies were resolved through discussion.

Data items

Data extraction was performed independently by two authors (M. G. and Y. Z.). Study characteristics, including bibliographic details (author, year), study design (type, control group, participant characteristics), and facility information (robot system, implant system, and control group system) were collected. Control groups included sCAIS and dCAIS, and study designs comprised clinical, in vitro, and animal studies. The deviation parameters recorded were: Global platform deviation (distance between planned and actual implant at the platform), global apex deviation (distance at the apex), and angular deviation (angle between planned and actual implant) as shown in Supplement 1. (http://www.medicinaoral.com/carpeta/suppl1_27132).

Risk of bias assessment

Two authors (J. W. and M. G.) independently assessed the risk of bias for each study using domain-based evaluation tools. The tools used included the Cochrane risk-of-bias tool for randomized trials (RoB 2) (12), the ROBINS-I for non-randomized studies (13), and SYRCLE's risk of bias tool for animal studies (14). The RoB 2 tool assessed RCT or controlled clinical trials (CCT) across five domains, while ROBINS-I assessed all retrospective studies (RS) across seven domains, and SYRCLE's tool evaluated animal studies across ten domains. The Quality Assessment Tool for In vitro Studies (QUIN Tool) (15) uses ten scales for assessment.

Bias categorization was based on the tool results: RCT/CCT studies were categorized as low, high, or unclear risk; RS as low, moderate, or critical risk; and in vitro studies as low, medium, or high risk. For animal studies, signaling questions guided bias judgments, with "yes" indicating low risk, "no" high risk, and "unclear" for uncertain risk. Results were presented graphically using the 'robvis' R package (16), including summary bar plots and traffic light plots illustrating domain-level risk of bias for each study.

Synthesis methods

Meta-analysis was performed using Review Manager (RevMan) software (version 5.3). A random-effects model was applied, with heterogeneity assessed using Cochrane's Q and I² tests (17). Statistical significance was defined as a p value&lt;0.05 for the Q test or I²&gt;50% for the I² test. Forest plots were generated to visually present the meta-analysis results. The mean difference (MD) was calculated using inverse variance for continuous outcomes, and a 95% confidence interval (CI) was computed. Meta-analysis was not performed for groups with only one study. Results were reported separately for sCAIS and dCAIS groups, considering global platform deviation, global apex deviation, and angular deviation measurements. Subgroup analysis was conducted when at least ten studies were available. Sensitivity analysis was performed by excluding studies with high risk of bias or imbalanced sample sizes, or by repeating the synthesis using a fixed-effects model.

Reporting bias assessment

If at least ten studies were included in the meta-analysis and heterogeneity was deemed acceptable (I²&lt;75%), a funnel plot and Egger's test for asymmetry would be used to assess publication bias.

Certainty of evidence

Given the study design, the GRADE approach (18) for assessing evidence certainty was not applicable.

## Results

Results

Study selection

A systematic review of literature was conducted across multiple electronic databases: PubMed (n=116), Embase (n=57), Cochrane Library (n=1), and CNKI (n=6), resulting in 180 initial records. A further manual search of reference lists from recent systematic reviews and meta-analyses (8 , 10 , 19 , 20) added one relevant study (21), bringing the total to 181 articles.

**Figure 1:**
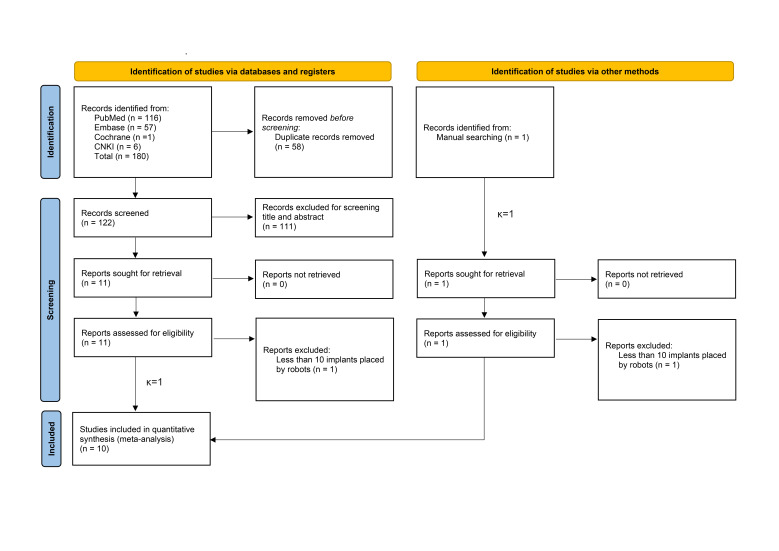
PRISMA 2020 flow diagram.

After removing duplicates, 123 records remained for screening. Of these, 111 records were excluded for not being robot-related or lacking accuracy measurements and comparisons. Eleven articles (22 - 32) and one additional manually searched article (21) were evaluated for eligibility. Two articles (21 , 22) were excluded for reporting fewer than ten implants placed by rCAIS. This left ten articles (23 - 32) for final inclusion. The flow diagram (Figure 1) illustrates the selection process, with Cohen kappa statistic (=1) indicating perfect agreement. Notably, the article by He et al. (26) reported both an in vitro study and a retrospective study, treated as separate studies - 'He et al. (a)' for the in vitro study, and 'He et al. (b)' for the retrospective study. Therefore, eleven studies were included in this systematic review.

1

Study characteristics

Characteristic information collected from the included studies was elucidated in Table 2. The studies included in this review were diverse in design: One CCT (29), four RS (26 , 27 , 30 , 32), five in vitro studies (24 - 26 , 28 , 31), and one animal study (23). Four clinical studies (26 , 27 , 29 , 32), two in vitro studies (26 , 31), and one animal study (23) compared rCAIS with sCAIS, while one clinical study (30) and three in vitro studies (24 , 25 , 28) compared rCAIS with dCAIS. All studies using sCAIS employed fully guided surgeries (23 , 26 , 27 , 29 , 31 , 32). To clarify the degree of automation, each included study was assessed and categorized according to a standardized robotic autonomy framework ranging from Level 0 to Level 5 (33).

Table 2

Risk of bias in studies

Risk of bias was assessed using appropriate tools for each study type. A summary of bias assessments was presented in Supplement 1. (http://www.medicinaoral.com/carpeta/suppl1_27132). The CCT (29) was at high risk of bias. Among the RS studies, one (26) was low risk, two (27 , 32) had moderate risk, and one (30) had serious risk. All in vitro studies (24 - 26 , 28 , 31) were assessed as low risk of bias.

Results of Syntheses

Two separate meta-analyses were conducted: One comparing rCAIS with dCAIS based on three in vitro studies (24 , 25 , 28), and another comparing rCAIS with sCAIS based on four clinical studies (26 , 27 , 29 , 32). Although meta-analysis was not feasible for certain comparisons due to limited data, a qualitative review of the available studies was conducted (Table 3). Sensitivity analyses confirmed the robustness of these results.

Table 3

**Figure 2:**
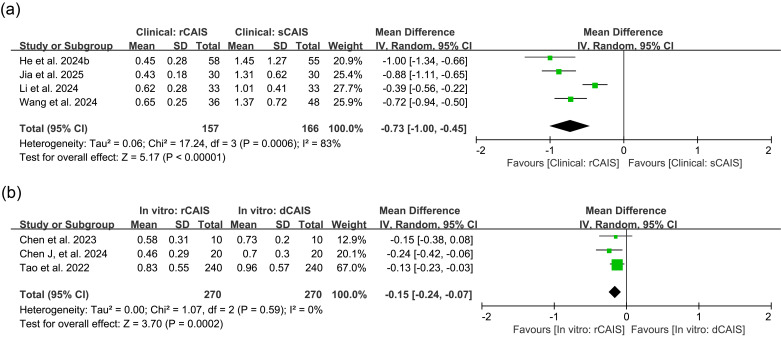
Comparison of global platform deviation. (a) Forest plot for rCAlS versus sCAlS in clinical studies; (b) Forest plot for rCAlS versus dCAlS in in vitrostudies. dCAIS: dynamic computer-assisted implant surgeries, IV: Inverse variance, rCAIS: robotic computer-assisted implant surgeries, sCAIS: static computerassisted implant surgeries, SD: Standard deviation.

For clinical studies comparing rCAIS and sCAIS in global platform deviation, the mean difference (MD) was -0.73mm (95% CI: [-1.00,-0.45], I2=83%, p&lt;0.00001). Comparisons of rCAIS and dCAIS in preclinical studies showed an MD of -0.15mm (95% CI: [-0.24,-0.07], I2=0%, p=0.0002), as presented in Figure 2.

2

Additionally, four studies excluded from meta-analysis reported platform deviation outcomes. In an in vitro study, He (a) et al. (26) compared rCAIS and sCAIS, observing platform deviation values of 0.58±0.60mm for rCAIS versus 1.50±1.46mm for sCAIS. Mozer et al. (31) conducted another in vitro study, reporting higher platform deviation in rCAIS (1.51±0.53mm) compared to sCAIS (0.79±0.28mm). In an animal study, Bai et al. (23) found that rCAIS exhibited a smaller platform deviation (0.269±0.152mm) than sCAIS (0.910±0.872mm). Furthermore, a retrospective study by Zhang et al. (30) comparing rCAIS with dCAIS reported platform deviation of 0.68±0.36mm in the rCAIS group and 1.25±0.54mm in the dCAIS group.

**Figure 3:**
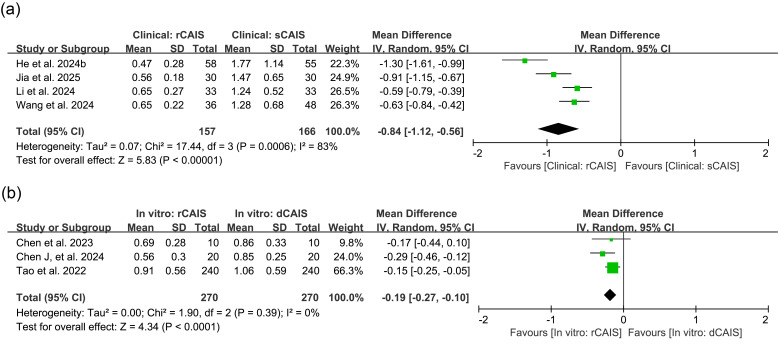
Comparison of global apex deviation. (a) Forest plot for rCAlS versus sCAlS in clinical studies;(b) Forest plot for rCAlS versus dCAlS in in vitro studies. dCAIS: dynamic computer-assisted implant surgeries, IV: Inverse variance, rCAIS: robotic computer-assisted implant surgeries, sCAIS: static computer-assisted implant surgeries, SD: Standard deviation.

In clinical studies assessing rCAIS versus sCAIS, the MD in global apex deviation was -0.84mm (95% CI: [-1.12,-0.56], I2=83%, p&lt;0.00001). Preclinical studies comparing rCAIS with dCAIS showed an MD of -0.19mm (95% CI: [-0.27, -0.10], I2=0%, p&lt;0.0001) (Figure 3). Studies excluded from the meta-analysis also provided data on global apex deviation. He (a) et al. (26) reported in vitro apex deviation of 0.58±0.60mm for rCAIS and 1.78±1.35mm for sCAIS. However, Mozer et al. (31) observed higher apex deviation in rCAIS (1.97±0.79mm) than in sCAIS (0.82±0.26mm) in vitro. In the animal experiment, Bai et al. (23) found that rCAIS had a smaller apex deviation (0.254±0.218mm) compared to sCAIS (1.179±1.176mm). Zhang et al.'s (30) retrospective study reported apex deviation of 0.69±0.36mm in rCAIS and 1.39±0.52mm in dCAIS.

3

**Figure 4:**
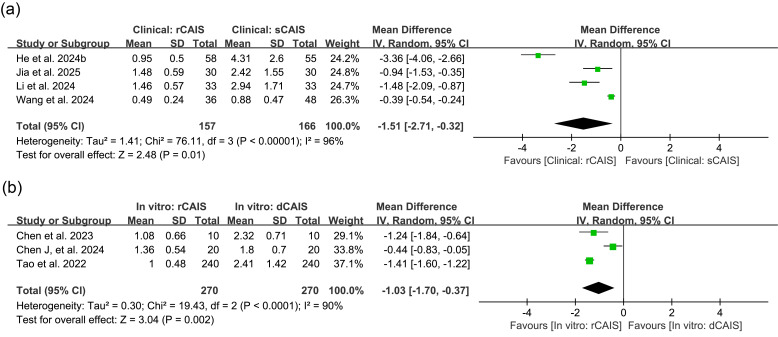
Comparison of angular deviation. (a) Forest plot for rCAlS versus sCAlS in clinical studies; (b) Forest plot for rCAIS versus dCAlS in in vitro studies. dCAIS: dynamic computer-assisted implant surgeries, IV: Inverse variance, rCAIS: robotic computer-assisted implant surgeries, sCAIS: static computer-assisted implant surgeries, SD: Standard deviation.

Clinical studies comparing rCAIS and sCAIS revealed an MD in angular deviation of -1.51° (95% CI: [-2.71,-0.32], I2=96%, p=0.01). For preclinical studies comparing rCAIS with dCAIS, the MD was -1.03° (95% CI: [-1.70,-0.37], I2=90%, p=0.002) (Figure 4). Studies not included in the meta-analysis reported similar patterns in angular deviation. He (a) et al. (26) documented in vitro angular deviation of 1.01±0.87° for rCAIS and 2.93±1.59° for sCAIS. Mozer et al. (31) observed higher angular deviation in rCAIS (2.66±1.83°) than in sCAIS (0.68±0.38°) in their in vitro model. Bai et al.'s (23) animal study showed that rCAIS had a smaller angular deviation (0.989±0.517°) compared to sCAIS (4.209±5.208°). In the retrospective study, Zhang et al. (30) reported angular deviation of 1.37±0.92° in rCAIS and 4.09±1.79° in dCAIS.

4

## Discussion

This study demonstrated a significant improvement with CAIS in implant positioning accuracy including reduced global platform, apex, and angular deviations. The findings reject the null hypothesis and confirm the superior accuracy of rCAIS in implant placement, providing a more comprehensive evaluation of rCAIS compared to sCAIS or dCAIS.

The advantages of rCAIS over sCAIS in terms of linear and angular errors can be attributed to several factors. Static guides tend to produce higher apex deviations due to the pivot point during osteotomy, whereas haptic robotic guidance in rCAIS, which lacks a pivot point, helps reduce apex deviations (34). Furthermore, rCAIS allows real-time adjustments to treatment plans, with continuous tracking of patient movement and immediate feedback to the surgeon, ensuring accurate implant placement. The robotic arm in rCAIS also mitigates the impact of surgeon hand tremors, reducing horizontal and angular deviations (5).

However, the study by Mozer et al. (31), which compared rCAIS with sCAIS in vitro, showed conflicting results. This discrepancy could be due to the use of a level 1 autonomy robot in Mozer et al.'s study, compared to level 2 robots in the other studies included in our analysis, as noted by Xu et al. (35). Additionally, two studies in this review highlighted specific challenges: Chen J, et al. (24) observed reduced accuracy in fresh extractions with rCAIS, possibly due to variations in bone density leading to mechanical arm slippage, while Chen et al. (25) found no significant differences between rCAIS and dCAIS in terms of platform and apex deviation, potentially due to the small sample size.

Regarding the superiority of rCAIS over dCAIS, the haptic feedback and physical guidance from the robotic arm in rCAIS enhance surgical stability. Unlike dCAIS, which requires timely adjustments during surgery, rCAIS preserves intraoperative stability and predictability. This human-robot collaboration allows for dynamic treatment planning and modification during surgery, further enhancing the precision of implant placement.

The bias assessment results showed commonalities, particularly regarding the inherent challenges of blinding in implant surgery (29). Given these challenges, some risks related to blinding are unavoidable. In vitro studies exhibited the lowest bias risk, likely due to careful sample size calculation and randomization techniques. In contrast, the animal study (23) had a more mixed bias profile, with one high-risk, four unclear-risk, and five low-risk ratings across ten domains using SYRCLE's tool. Notably, the high-risk assessment was attributed to inadequate generation and application of the allocation sequence. Among the clinical studies, the CCT (29) had a high risk of bias due to the lack of randomization in group allocation. Two RS studies (27 , 32) were rated as moderate risk due to insufficient information on whether outcome assessors were blinded to the interventions. Another RS by Zhang et al. (30) had a serious risk of bias due to improper handling of confounding variables and missing outcome data.

Regarding heterogeneity, the clinical studies comparing rCAIS with sCAIS showed significant heterogeneity (I²&gt;50%), likely due to the mix of study types, including one CCT and three RS. In contrast, the in vitro studies comparing rCAIS with dCAIS showed lower heterogeneity (I²=0%) for global platform and apex deviations. However, the angular deviation group showed high heterogeneity (I²=90%) ; sensitivity analysis identified that the heterogeneity was influenced by the study by Chen et al. (25). The high variability may also arise from differences in dentition states among participants. Given the limited number of studies in this meta-analysis, conducting a subgroup analysis to assess the impact of edentulism was not feasible. Nevertheless, a prior systematic review (19) suggests that variations in edentulism types and robotic systems likely have a negligible impact on results. Additionally, the use of fully-guided protocols across all included studies (26 , 27 , 29 , 32) minimizes concerns regarding differences in guidance methods, given that no significant difference was observed between fully and partially guided protocols in previous studies (9).

This present systematic review distinguishes itself from previous studies by implementing significantly stricter inclusion criteria. Unlike prior systematic reviews that included single-cohort investigations of robotic systems (e.g., Khan et al. (20)), our systematic review exclusively focused on studies with direct head-to-head evaluations of rCAIS and sCAIS/dCAIS, thereby ensuring a higher level of methodological rigor and more robust comparative evidence. Moreover, our meta-analysis separately synthesized data from both clinical and model studies, offering a more nuanced understanding of the performance differences between rCAIS and sCAIS/dCAIS. These approaches not only enhance the reliability of our findings but also contribute novel insights that extend the current literature in this field. In addition, the previous study by Khan et al. (21) reported that rCAIS demonstrated greater accuracy in terms of coronal, apical, and angular deviations compared with dCAIS, with MD of -0.17mm (95% CI: [-0.24,0.09], p&lt;0.001), -0.21mm (95% CI: [-0.36,-0.06], p=0.006), and -1.41° (95% CI: [-1.56,-1.26], p&lt;.001). These results were similar and consistent with the present study.

Despite the promising accuracy benefits of rCAIS demonstrated in this study, several limitations and challenges must be considered. First, the substantial financial investment required for robotic equipment acquisition and maintenance may restrict its widespread adoption. Furthermore, the preoperative preparation for robotic systems is relatively lengthy and complex. As demonstrated by Bai et al. (23) in their comparative study, rCAIS procedures required longer operative durations than sCAIS. Third, most current robotic platforms lack haptic feedback, which can impair the surgeon's tactile perception and result in a preference toward the lower-density bone side during osteotomy (36). These limitations collectively highlight the gap between the technical precision of rCAIS and its practical integration into routine clinical workflows, necessitating further exploration of strategies to address these challenges.

A notable limitation of this study is its reliance CCT and RS, which precluded the application of the GRADE system to assess the certainty of evidence (18). Future research should incorporate more high-quality randomized controlled trials. Additionally, a meta-analysis of preclinical studies comparing sCAIS and rCAIS was not feasible due to the limited number of eligible studies, as well as heterogeneity in robotic autonomy levels and study designs (23 , 26 , 31). Therefore, future preclinical investigations employing standardized protocols and comparable robotic platforms are needed to support meaningful meta-analyses.

## Conclusions

This systematic review demonstrates that rCAIS achieves greater implant placement accuracy compared to both sCAIS and dCAIS. However, the magnitude of observed differences is small, and thus these improvements may not be clinically relevant despite the statistical significance. Further well-designed and large-scale clinical trials are needed to clarify the factors influencing these outcomes and optimize the application of robotic systems in implantology.

## Figures and Tables

**Table 1:** Table Search strategy.

Database	Combination of Search Terms and Strategy		N*
PubMed	#1	Dental Implants [MeSH Terms]	116
	#2	dental	
	#3	implant	
	#4	#2 AND #3	
	#5	#1 OR #4	
	#6	Robotic Surgical Procedures [MeSH Terms]	
	#7	robot	
	#8	assist	
	#9	guide	
	#10	#8 OR #9	
	#11	#7 AND #10	
	#12	#6 OR #11	
	#13	accuracy	
	#14	precision	
	#15	#13 OR #14	
	#16	#5 AND #12 AND #15	
Embase	#1	tooth implant/exp	57
	#2	dental	
	#3	implant	
	#4	#2 AND #3	
	#5	#1 OR #4	
	#6	robot assisted surgery/exp	
	#7	robot	
	#8	assist	
	
	#9	guide	
	#10	#8 OR #9	
	#11	#7 AND #10	
	#12	#6 OR #11	
	#13	accuracy	
	#14	precision	
	#15	#13 OR #14	
	#16	#5 AND #12 AND #15	
Cochrane Library	#1	MeSH descriptor: [Dental Implants] explode all trees	1
	#2	MeSH descriptor:[Robotic Surgical Procedures] explode all trees	
	#3	accuracy	
	#4	precision	
	#5	#3 OR #4	
	#6	#1 AND #2 AND #5	

*N: Number of retrieved articles.

**Table 2:** Table Characteristics of including studies.

Study	Year	Language	Study Type	Control group	State of dentition	Jaw distribution	Robot system	Autonomous level	Implant system	Control group system	Implant placement protocol
Tao et al. [28]	2022	English	In vitro	dCAIS	Partially/ fully edentulous	Maxilla and mandible	Hybrid Robotic System for Dental Implant Surgery(Shanghai, China)	2	4.1 x 10mm; Straumann SP	Beidou	Delayed
Chen et al. [25]	2023	English	In vitro	dCAIS	Partially edentulous	Maxilla	THETA robotic dental implant system (Hangzhou Jianjia Robot, Ltd.)	2	Mixed sizes (diameters:3.5/4.3/5.0 mm; lengths: 8/10/11.5mm);NobelParallelConical Connection	Yizhimei	Delayed
He et al. (a) [26]	2024	English	In vitro	sCAIS	Single tooth	Maxilla	Dental Navi (YakeRobot Technology, Ltd.)	2	3.3 x 10mm; Straumann BLT	Digital implant guide	NA
He et al. (b) [26]	2024	English	RS	sCAIS	Partially edentulous	NA	Dental Navi (YakeRobot Technology, Ltd.)	2	Mixed sizes (diameters:3.3/4.1/4.8 mm; lengths:8/10/12/14/16mm);Straumann BL/BLT	Digital implant guide	NA
Chen J, et al. [24]	2024	English	In vitro	dCAIS	Single tooth	Maxilla and mandible	Image-Guided AutonomousRobot Dental Surgery System(Remebot, Baihui Weikang Technology, Ltd.)	2	4.1 x 10mm; Straumann BLT	Yizhimei	Immediate and delayed
Jia et al. [27]	2025	English	RS	sCAIS	Partially edentulous	Maxilla and mandible	ADIR system (Xian, China)	2	Straumann BLX	Digital implant guide	Delayed
Bai et al. [23]	2021	Chinese	Animal	sCAIS	Partially edentulous	Mandible	ADIR system (Xian, China)	2	4.1 x 12mm; Straumann	Digital implant guide	Delayed
Zhang et al. [30]	2024	English	RS	dCAIS	Partially edentulous	Maxilla and mandible	Image-Guided AutonomousRobot Dental Surgery System(Remebot, Baihui Weikang Technology, Ltd.)	2	Multiple implant systems	Dental Implant Navigation System	NA
Wang et al. [29]	2024	English	CCT	sCAIS	Edentulous	Maxilla and mandible	Dental Navi (YakeRobot Technology, Ltd.)	2	Straumann BL	Digital implant guide	Immediate
Mozer et al. [31]	2024	English	In vitro	sCAIS	Partially edentulous	Maxilla and mandible	Yomi (Neocis, Inc.)	1	NobelActive(5.1 x 10 mm,4.3 x 11.5 mm,3.5 x 10 mm)	Digital implant guide	Delayed
Li et al. [32]	2024	English	RS	sCAIS	Partially edentulous	Maxilla and mandible	Image-Guided AutonomousRobot Dental Surgery System(Remebot, Baihui Weikang Technology, Ltd.)	2	NA	Digital implant guide	Immediate

BL: Bone level, BLT: Bone level tapered, CCT: Controlled clinical trial, dCAIS: Dynamic computer-assisted implant surgeries, NA: Not available, RS: Retrospective study, mm: Millimeter, sCAIS: Static computer-assisted implant surgeries, SP: Standard plus.

**Table 3:** Table Conclusions of included studies.

Study	Implants (N)	Conclusion
Tao et al. [28]	N=480	rCAIS showed significantly lower entry, exit and angle deviations compared to dCAIS (p<0.05)
Chen et al. [25]	N=20	rCAIS (THETA) showed significantly better angular accuracy than dCAIS (Yizhimei), with no significant difference in coronal and apical deviation
He et al. (a) [26]	N=80	rCAIS showed significantly higher accuracy in coronal, apical and angular deviation than sCAIS (p<0.001)
He et al. (b) [26]	N=113	rCAIS showed significantly higher accuracy than sCAIS in all 3D deviation parameters (p<0.001)
Chen J et al. [24]	N=40	rCAIS showed significantly higher angular accuracy in both fresh and healed sites and better coronal/apical accuracy at healed sites (p<0.05)
Jia et al. [27]	N=60	rCAIS showed significantly higher accuracy than sCAIS in all deviation parameters (p<0.01)
Bai et al. [23]	N=36	In vivo animal study showed significantly greater accuracy with rCAIS compared to sCAIS in all parameters (p<0.001)
Zhang et al. [30]	N=124	rCAIS showed significantly higher accuracy than dCAIS in angular, coronal and apical deviations (p< 0.001)
Wang et al. [29]	N=84	rCAIS showed significantly greater accuracy in all deviation types compared to sCAIS (p< 0.01)
Mozer et al. [31]	N=102	sCAIS showed significantly better accuracy than rCAIS in all parameters (p<0.001)
Li et al. [32]	N=66	rCAIS demonstrated significantly better coronal, apical and angular accuracy than sCAIS in immediate anterior implantation (p< 0.05)

dCAIS: Dynamic computer-assisted implant surgeries, M: Median, Q: Quartile, rCAIS: Robotic computer-assisted implant surgeries, sCAIS: Static computer-assisted implant surgeries.

## Data Availability

The datasets used and/or analyzed during the current study are available from the corresponding author.
